# Clathrate Adhesion Induced by Quasi-Liquid Layer

**DOI:** 10.1021/acs.jpcc.1c06997

**Published:** 2021-09-16

**Authors:** Ngoc N. Nguyen, Rüdiger Berger, Michael Kappl, Hans-Jürgen Butt

**Affiliations:** † Physics at Interfaces, Max Planck Institute for Polymer Research, Ackermannweg 10, 55128 Mainz, Germany; ‡ School of Chemical Engineering, Hanoi University of Science and Technology, Dai Co Viet Street 1, Hanoi 100000, Vietnam

## Abstract

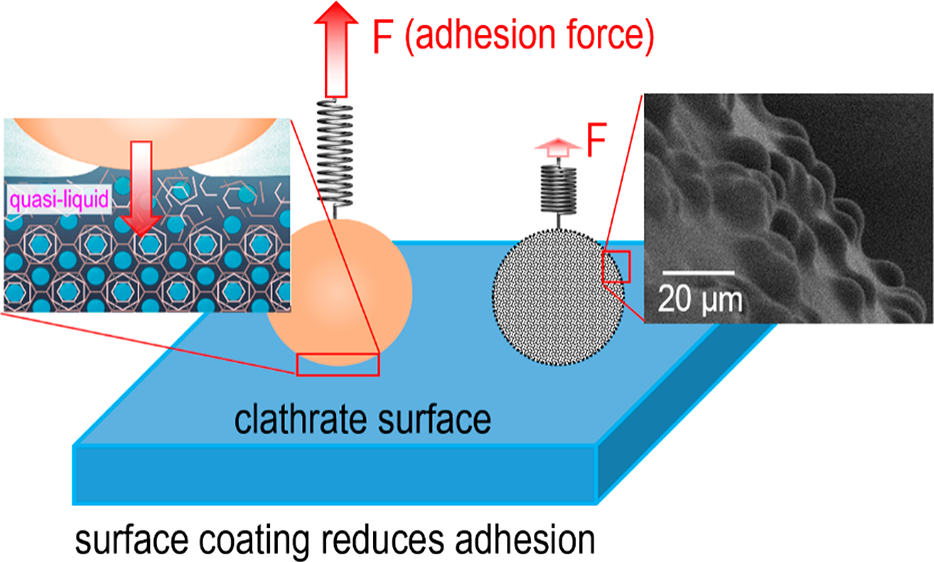

The adhesive force of clathrates to surfaces is a century-old problem of pipeline blockage for the energy industry. Here, we provide new physical insight into the origin of this force by accounting for the existence of a quasi-liquid layer (QLL) on clathrate surfaces. To gain this insight, we measure the adhesive force between a tetrahydrofuran clathrate and a solid sphere. We detect a strong adhesion, which originates from a capillary bridge that is formed from a nanometer-thick QLL on the clathrate surface. The curvature of this capillary bridge is nanoscaled, causes a large negative Laplace pressure, and leads to a strong capillary attraction. The microscopic capillary bridge expands and consolidates over time. This dynamic behavior explains the time-dependent increase of measured capillary forces. The adhesive force decreases greatly upon increasing the roughness and the hydrophobicity of the sphere, which founds the fundamental basics for reducing clathrate adhesion by using surface coating.

## 1 Introduction

Adhesive forces between clathrates and solid surfaces are a field of practical importance.^1−6^ Especially, this force causes a century-old problem of clathrate agglomeration and the consequence of pipeline blockage in the oil and gas industry. However, the origin of adhesion forces for clathrate surfaces is poorly understood. Recently, it was reported that clathrate surfaces are not rigid solids but covered by an intrinsic nanometer-thick quasi-liquid layer (QLL) due to surface premelting.^7−11^ However, to what extent the QLL affects the adhesion force of clathrate surfaces was not understood. Therefore, we carry out force measurements and theoretical modeling to find out how the QLL affects the adhesion of clathrate surfaces to other solid surfaces.

Fundamentally, clathrates are solid structures composed of water and guest molecules.^12−14^ The structures are sustained by the hydrogen bonding of water molecules that leads to an ice-like framework of water which acts as a host structure. Guest molecules are incorporated in the regular cavities of the host structure.^15^ In this way, clathrate structures encapsulate guest molecules up to mole fractions of an order of 0.1, although most guest species are hydrophobic and nearly insoluble in liquid water.^15^ In nature, the largest part of methane on Earth is encapsulated in natural clathrates under subsea and permafrost sediments.^15,16^ Natural clathrates constitute a vast source of low-carbon energy but also a significant environmental hazard due to potential large-scale emissions of methane as a potent greenhouse gas.^16,17^ Meanwhile, synthetic clathrates are considered as the next generation material for fuel gas storage.^18−23^ For example, hydrogen can be loaded into a hosting clathrate formed by tetrahydrofuran (THF) up to a mass fraction of 4.0 wt %.^24^ High storage capacity and slow liberation of gas upon dissociation enable efficient and safe storage of fuel gas based on clathrates.^24,25^ Nevertheless, clathrates are a major safety hazard in the oil and gas industry. Residual water present in crude oil or natural gas tends to form clathrate particles which then accumulate at the pipe surfaces and plug the flow.^26−28^

For all fields mentioned above, the adhesive force between clathrates and natural or technical surfaces plays a significant role. This force drives the adhesion of clathrate particles to pipe surfaces in oil and gas pipelines and leads to plugging.^26−28^ Similar problems happen in the facilities for clathrate productions, e.g., for storage of fuel gas. Synthetic clathrates tend to attach to equipment walls, accumulate, and finally block the reactors and circulation systems. Natural clathrates contact and interact with a variety of natural solid matters through clathrate-solid interfaces.^29−31^ The interaction at the interfaces influences the mechanical and physiochemical stability of the clathrate deposits. Thus, a better understanding of adhesion forces is required for exploiting natural clathrates.

Here, we outline the physics of adhesion forces induced by a nanometer-thick QLL on clathrate surfaces. Our calculations are in good agreement with measured adhesion forces acting between a flat clathrate surface and a solid sphere. Using the configuration of a flat clathrate surface interacting with a well-defined, spherical solid particle, we are able to assess the role of the nanoscale QLL for the adhesion. In applied systems, usually clathrate particles interact with flat surfaces such as the pipeline wall. However, preparation of clathrate particles with smooth, well-defined surfaces is hardly achievable.^1,2,32,33^ The arbitrary shapes of clathrate particles would complicate the theoretical analysis of the adhesive forces. More importantly, the adhesive force between a solid sphere and a flat clathrate surface is comparable to the force between a clathrate sphere and a flat solid surface (Supporting Information). Furthermore, we explore the expansion and consolidation of the QLL-formed capillary bridge between the clathrate surface and the solid sphere, which leads to solid–solid adhesion and increased adhesive force at prolonged contact times. On the basis of the capillary nature of the adhesion mechanism, we develop a simple and efficient coating with defined roughness and hydrophobicity for reducing the clathrate adhesion.

## 2 Experimental and Theoretical Methods

### 2.1 Experiments

#### Preparation of Clathrate Surfaces

**Figure 1:**
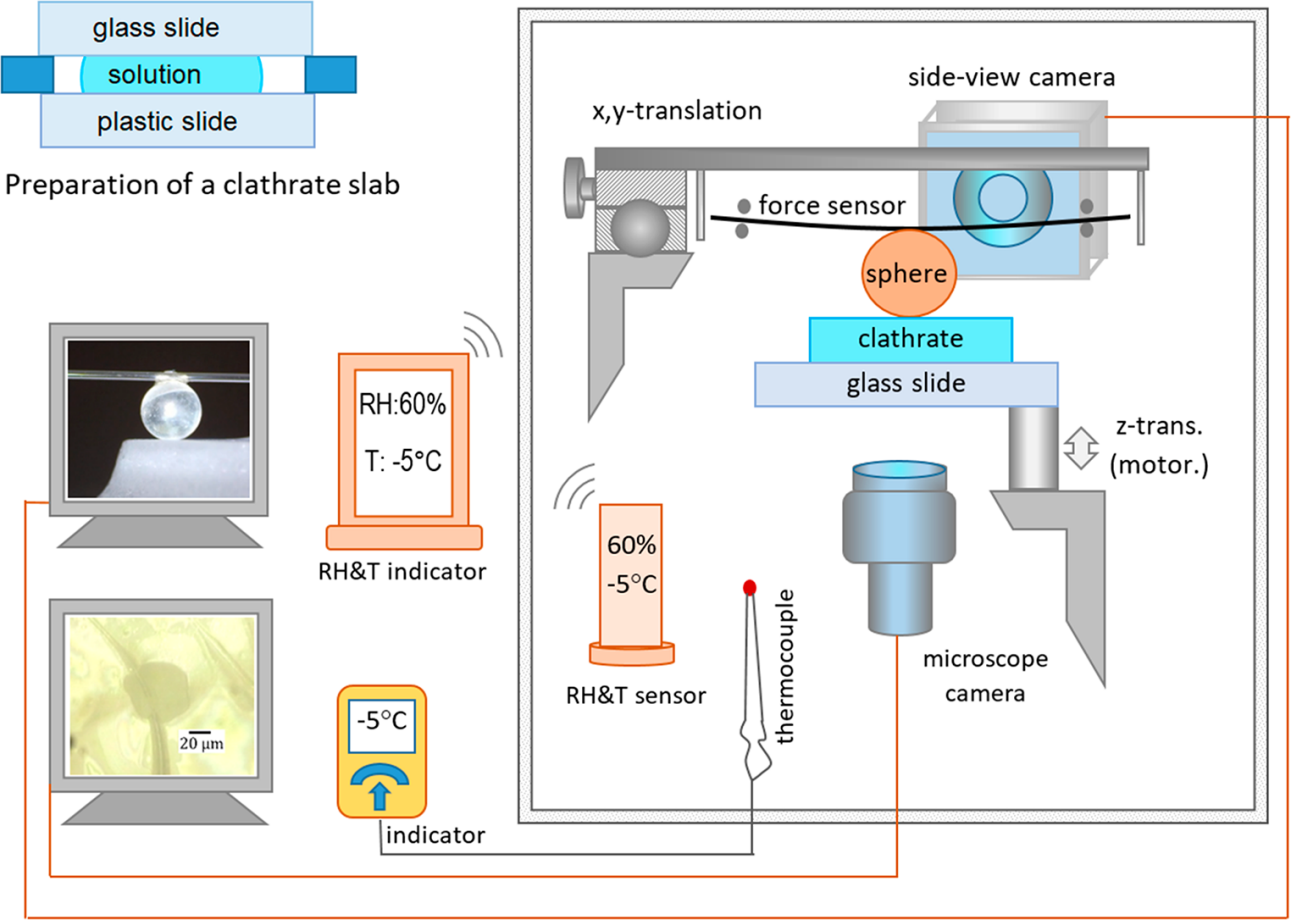
Setup for the measurement of adhesive force and the probe of the expansion of the contact area. Experiments were carried out in a cold box (details provided in the Supporting Information).

We used the clathrate of deuterated tetrahydrofuran (THF-*d*_8_, C_4_D_8_O 99.5 atom % D, Carl Roth GmbH) because we used the same stock solution for nuclear magnetic resonance (NMR) experiments.^34^ The independent NMR experiments were performed in parallel addressing the origin of phase transitions in clathrates.^34^ Hereafter, the THF-*d*_8_ clathrate may be simply referred to as “clathrate”. Clathrates were prepared in a freezer at atmospheric pressure through the following steps. A volume (70 μL) of a stoichiometric solution (mole ratio: ) was deposited on a plastic slide. A glass slide was imposed in parallel to the plastic one to confine the solution in the gap (Figure 1). The sample was then cooled down to −15 °C in a freezer. Finally, the plastic surface was removed and a clathrate slab remained on the glass slide.

**Figure 2:**
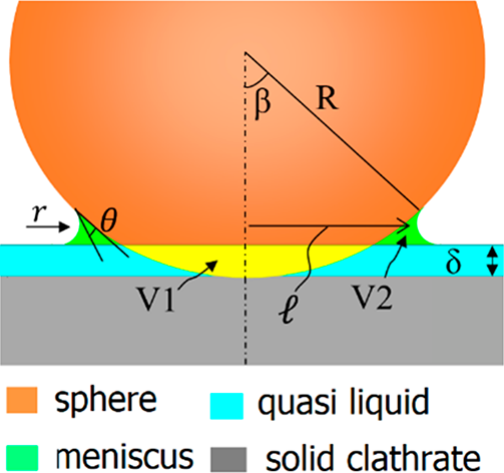
Model of the resulting nano capillary bridge (green domain) which induces an adhesive force between the sphere and clathrate surface. Drawing is not to scale.

#### Coating of Silica Spheres

We tuned the hydrophobicity of the silica spheres (*R* = 0.8 mm) via adding a coating to it. The original silica sphere has a contact angle (θ) around 10° with stoichiometric THF-*d*_8_ solution. Silica spheres were coated in three ways. Gold coating: a 20 nm-thick layer of gold was coated on the surface of silica spheres using the sputter method (BAL-TEC, MED 020 Coating System) under argon plasma at 10^–5^ at. Teflon coating: Teflon has been identified as having very low free surface energies and low adhesive properties.^35^ A silica sphere was dip-coated in a solution of 4 wt % Teflon AF1600 in FC-43 solvent (Sigma-Aldrich) for 1 min. After removing the sphere from the solution, it was annealed at 160 °C for 12 h for complete removal of the solvent and consolidation of the Teflon film. It resulted in a stable Teflon film of 400 nm thickness covering the silica sphere. Rough hydrophobic coating: Microspheres (mean diameter 10 μm, Bangs Laboratories Inc.) were dispersed in the aforementioned Teflon solution to 5 wt % concentration by shaking and 30 min sonication. Then, the coating was implemented through the same dip-coating procedure as described for Teflon coating. The surface of the silica sphere (*R* = 0.8 mm) was coated by microspheres who themselves were coated by a Teflon film. These three methods led to contact angles of 62°, 115°, and 140° with the solution of dissociated THF clathrate, respectively. Details about coating and contact angle measurements are discussed in the Supporting Information.

#### Measurement of the Adhesive force

We developed a home-built setup for measuring the adhesive force between a clathrate surface and a solid sphere (Figure 1). Hollow microcapillary glass tubes were used as mechanical force sensors^36,37^ (Figure S3). Each sphere was glued to the middle of a force sensor using epoxy glue. Having the sphere positioned in the center of the force sensor while both sides of the force sensor can move freely in a horizontal direction avoids any sliding motion of the sphere relative to the clathrate surface (Figure 1). This improved feature provides a better accuracy of the force measurements (Figure S5). We used two different geometries of force sensors, which allowed measuring the minimum forces of 0.7 μN and 3.5 μN, respectively (Figure S3). The *x*,*y*-translation stages were used to move the sphere laterally on the clathrate surface for measurements at different locations. A motorized z-translation stage engages the clathrate surface toward the sphere until it gently touches the sphere. After a defined contact time, the surface is withdrawn at a speed of 0.07 mm/s. The sphere adheres to the clathrate surface until the restoring force exerted by the force sensor exceeds the adhesive force. The adhesive force is determined in accordance to Hooke’s law: *F* = *k* × Δ*x*. Force sensors having a spring constant (*k*) of 0.45 N/m were used for the original silica spheres and the gold-coated spheres. Force sensors having *k* = 0.026 N/m were used for the Teflon-coated spheres and the rough hydrophobic spheres. Spring constants were measured by applying reference weights to the center point of the force sensors. The deflection of the force sensor, Δ*x*, is defined at the center of the capillary tube and measured using a side-view camera.

Experiments on clathrate surfaces were conducted in a home-built “cold box” having an internal volume of 2 m^3^. Sample and setup are handled with gastight gloves fixed to the transparent front side of the cold box (Figure S4). The temperature (*T*) and relative humidity (RH) were constantly monitored (KLIMALOGG Pro, TFA, accuracy of ±0.1 °C and ±1%), respectively. The temperature is confirmed by a parallel measurement with a thermocouple (±0.1 °C) and a multimeter (EX505, Extech Instruments). Details are provided in the Supporting Information.

#### Probing the Contact Area

This experimental setup is similar to the force measurement (Figure 1). A 0.3 mm thin clathrate slab is used for better transparency. The clathrate surface is driven vertically to touch the sphere, and the evolution of the contact area is recorded by a video microscope (Olympus) from below.

### 2.2 Modeling Capillary Forces

We propose a simple model to describe the physical origin of the adhesive force. Our model accounts for the premelting nature of clathrate surfaces by treating the premelting layer as a QLL on a solid clathrate substrate. As depicted in Figure 2, the approaching sphere displaces a volume of quasi liquid (*V*_1_, yellow region) and leads to the formation of a microscopic meniscus, i.e., nano capillary bridge (*V*_2_, green region). This displacement happens as soon as the sphere touches the surface of the QLL. For a short time after the attachment, the total volume of quasi liquid is considered constant, thus *V*_2_ = *V*_1_. The meniscus induces a capillary force (*F*_ca_) acting between the sphere and the clathrate surface.

Capillary force *F*_ca_ is the sum of the integral of the normal component of the interfacial tension around the neck (*F*_*γ*_) and the contribution of the Laplace pressure acting over the contact area (*F*_Δ*P*_).^38,39^
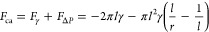
1

Here, the minus signs indicates an attractive force. γ, , and *r* are the interfacial tension of the quasi liquid and the two principal radii of curvature, respectively (Figure 2). For geometric reasons,  and *r* can be expressed as a function of the contact angle θ of the quasi liquid with the sphere surface (Supporting Information):
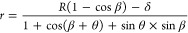
2
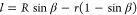
3

*R*, δ, and β are the sphere radius, thickness of the QLL, and the filling angle, respectively. In the initial phase, where the sphere has just penetrated the QLL but the meniscus has not yet grown much, the geometric parameters are constrained by the condition of *V*_1_ ≈ *V*_2_. Assuming *V*_1_ = *V*_2_, the parameters can be expressed by eq 4 (Supporting Information):
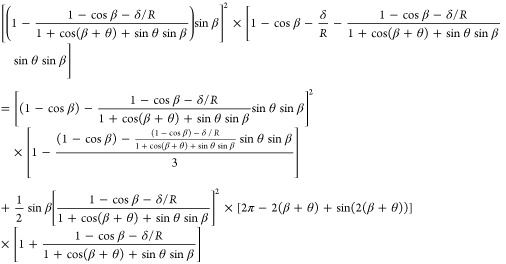
4

It is noted that eq 4 only contains β as a variable. Other parameters are constants for a given physical system (*R*, θ, and δ). The QLL thickness (δ) is a temperature-dependent parameter.^40−42^ However, quantitative data on the QLL on clathrate surfaces are very rare. Our recent experiments showed δ ≈ 10 nm for the QLL on the surface of semiclathrate formed by tetrabutyl ammonium bromide at −4 °C.^7^ This value (10 nm) agrees with typical thickness of QLL on pristine ice surfaces at temperatures of several minus degrees centigrade.^41,42^ Therefore, we used δ = 10 nm as a representative and constant value for the clathrate in our model, without considering a likely temperature dependence of the QLL thickness. To estimate the surface tension of the quasi liquid, we measured the surface tension of a dissociated THF clathrate at 4 °C and obtained γ = 33.5 mN/m. This value is used in our model.

Equation 4 can be solved numerically to obtain β, which is used for calculating , *r*, and *F*_ca_ in eq 1–3. In particular, we vary θ in a range between 10° and 160° to study the effect of the surface hydrophobicity of the solid on the capillary force.

## 3 Results and Discussions

### 3.1 Experimental Observations

**Figure 3:**
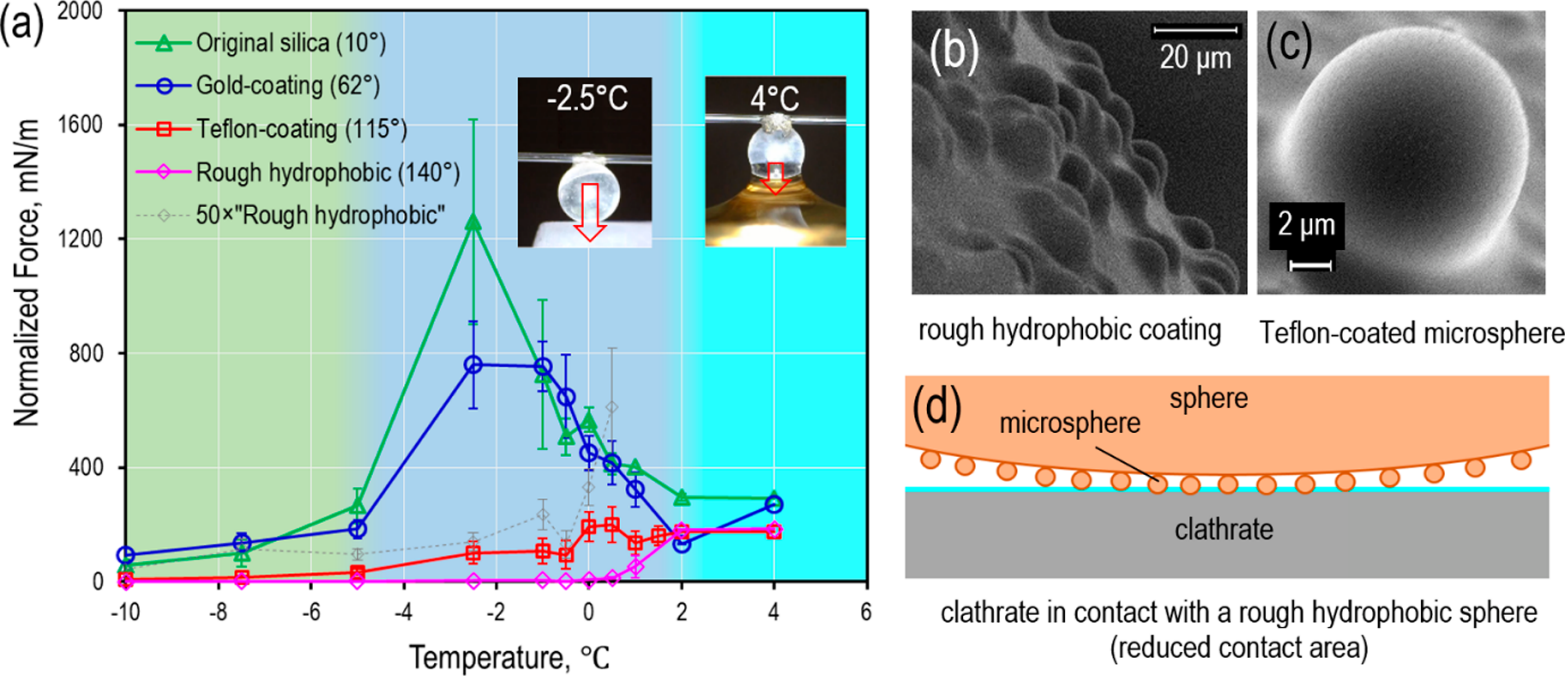
(a) Normalized adhesion force measured between a THF-*d*_8_ clathrate surface and a solid sphere (*R* = 0.8 mm) with different coatings and 0.5 min contact time. Inset images visualize the adhesion in the premelting region and the dissociated region for the experiments using an original silica sphere. Background colors of the graph indicate three distinct temperature regions disucssed in the text. (b) SEM image of the surface of a rough hydrophobic sphere coated by microspheres and a Teflon film on the top. (c) Close look at a Teflon-coated microsphere on the rough hydrophobic sphere. (d) Concept of the reduced contact area between a rough hydrophobic sphere and a clathrate surface (not to scale).

When measuring the adhesive force between the clathrate surface and solid spheres in dependence on temperature, three regions can be distinguished (Figure 3a). The measured forces were normalized by dividing the value by the radius of the sphere. For spheres with contact angle ≤ 115°, weak forces are observed at *T* < −5 °C. In this region of low temperatures, the QLL might vanish or become discontinuous so that the clathrate surface behaves like a typical solid that does not form a capillary bridge to the sphere. As a result, the adhesive forces are low. The second region spanning from −5.0 to 2.0 °C corresponds to the “premelting region” since the temperatures are below the dissociation point of the clathrate. This premelting region is associated by a 1 order of magnitude increase of the adhesive force (Figure 3a). We attribute such large adhesive forces to the formation of a nano capillary bridge between the clathrate surface and the sphere due to the QLL (section 2.2). Such nano capillary bridges cause capillary forces. For temperatures > 2.0 °C, named here as “dissociated region”, the capillary forces decrease substantially before staying at constant values (Figure 3a). The clathrate softened at 2.0 °C and dissociated gradually. Complete dissociation observed at 4.0 °C results in a THF-*d*_8_ solution interacting with the sphere (inset in Figure 3a). The present observation of gradual dissociation of THF-*d*_8_ clathrate agrees with our recent nuclear magnetic resonance experiments.^34^ The THF-*d*_8_ clathrate does not have a fixed dissociation point; instead, it dissociates gradually in the temperature range from 2 to 4 °C.^34^

Our experiments reveal that the capillary force between the clathrate surface and the hydrophilic sphere is largest in the premelting region. For the original silica sphere, the capillary force is on the order of 1000 mN/m. In this region, the QLL thickness is on the order of 10 nm.^7,41,42^ We recorded optical images close to the maximum force values in both premelting and dissociated regimes (insets in Figure 3a). For the dissociated clathrate at 4 °C, the liquid deforms visually over a large vertical range and creates a macroscopic capillary bridge. Despite that large meniscus deformation, the capillary force at 4 °C is an order of magnitude smaller than that at −2.5 °C. This finding is intriguing and is not reported so far in the literature although an order increase in adhesive force in the premelting region is of profound importance as mentioned in the introduction section.

The measured capillary forces decrease with increasing contact angle of the sphere (Figure 3a). In particular, the large capillary forces in the premelting region vanish for the rough hydrophobic (superhydrophobic-like) sphere. Only a low normalized force of 4.2 mN/m was observed instead. This low capillary force is ascribed to the reduced contact area of the rough hydrophobic sphere with the QLL on the clathrate surface as discussed below.

### 3.2 Origin of Large Capillary Forces in Premelting Region

**Figure 4:**
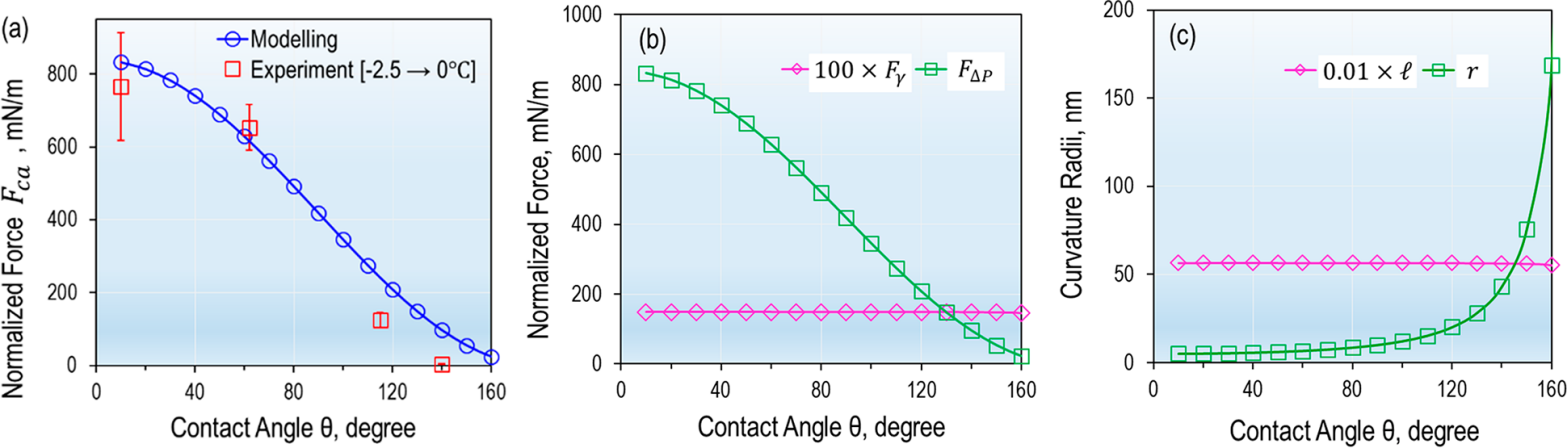
(a) Normalized calculated capillary force versus the experimental force. (b) The Laplace pressure force and the normal surface tension force. (c) The radii of curvature. These parameters were calculated using eqs 1–3. The plots of *F*_*γ*_ and  are scaled accordingly to fit the graphs. Each data point for the experimental force is the averaged force in the temperature region between −2.5 and 0.0 °C, taken from Figure 3a, contact time of 0.5 min.

In order to understand the force increase in the premelting region, we plotted the normalized capillary force () calculated from eq 1 for γ = 33.5 mN/m, δ = 10 nm, and *R* = 0.8 mm versus contact angle (Figure 4a). The calculated normalized capillary force decreases with increasing contact angle, i.e., increasing hydrophobicity of the sphere. For example, the normalized capillary force is 833 mN/m for a hydrophilic sphere (θ = 10°) but is reduced to 25 mN/m for a superhydrophobic sphere (θ = 160°).

The decrease of the calculated capillary force upon increasing the hydrophobicity of the sphere is in quantitative agreement with our experimental results (Figure 4a). Both the magnitude and the hydrophobicity-dependence of modeled forces are reproduced by experiments. For original silica spheres (10°) and gold-coated spheres (62°), the differences between experimental and modeled forces fall within the experimental errors (Figure 4a). Significant apparent discrepancy is observed only for the rough hydrophobic sphere (140°) where the modeled force is 97.4 mN/m while the measured force is only 4.2 mN/m. This discrepancy arises from the fact that the modeled force (97.4 mN/m) was calculated based on a smooth hydrophobic sphere of 0.8 mm radius and 140° contact angle. However, experiments were carried out on a sphere coated by 10 μm particles to form a rough sphere (Figure 3b,c) with effective contact angle of 140°. Note that this contact angle could not be achieved for any smooth surface.^43,44^ This rough sphere contacts the QLL only through a number of the microspheres (Figure 3d). Taking this contact configuration into account, we calculated the expected capillary force by applying our model to each microsphere contacting the QLL (Figure 3d). The calculated capillary force varies in a range between 2.8 and 8.6 mN/m (sum of capillary forces acting on microspheres contacting the QLL divided by the apparent radius of the big silica sphere), depending on the coverage (i.e., density) of the microspheres coated on the silica sphere. The calculated force falls in the same range of the measured force. Hence, the model provides a universal physical principle for describing the capillary force between a solid sphere and clathrate surface.

When comparing the contributions of the Laplace pressure force (*F*_ΔP_) and the surface tension force (*F*_*γ*_) to the total capillary force (*F*_ca_), we find that the *F*_ΔP_ dominates (Figure 4b). Moreover, *F*_ΔP_ is very sensitive to the hydrophobicity of the solid surface. This force decreases from 831 mN/m at θ = 10° to 23 mN/m at θ = 160°. In contrast, the surface tension force *F*_*γ*_ only decreases slightly from 1.5 to 1.4 mN/m for the same contact angle increase. Meanwhile, the radius of curvature *r* is 3 orders of magnitude smaller than the radius  (Figure 4c). For example, *r* increases strongly from 5 nm at θ = 10° to 168 nm at θ = 160°, whereas  only decreases slightly from 5.6 μm at θ = 10° to 5.5 μm at θ = 160°. The nanoscaled radius (*r*) of curvature gives rise to the dominating Laplace pressure force compared to contribution of the surface tension force (Figure 4b,c). In other words, the nanoscaled radius of curvature *r* is the determining factor of the capillary force at clathrate surfaces.

Incorporating the modeling results with the experimental force in Figure 3a leads to the following conclusions. First, the capillary force induced by a nano capillary bridge in the premelting region is large because of a small respective radius *r*. Second, the capillary force induced by macroscopic meniscus in the dissociated region is weak because of a much larger corresponding radius *r*. Third, the decrease in the capillary force upon approaching the temperature of the dissociated region can be explained by the rapid thickening of the QLL. In general, QLL thickens as the temperature increases^9,42^ and leads to an increasing radius *r*. Finally, low adhesive forces in the low-temperature region are explained by a vanishing or discontinuous QLL. The latter makes the clathrate surface behave solid-like, with only van der Waals forces acting.

In the experiments described so far, we have used a short contact time of 0.5 min. During this initial phase of the sphere-clathrate attachment, the nano capillary bridge forms and dominates the adhesive force between the two objects. The measured adhesive force is fully described by the capillary force in our model (Figure 4a) based on the approximation *V*_1_ ≈ *V*_2_. For longer contact times, the measured forces increase (discussed below) and exceed the forces predicted by our model. This suggests that the initial meniscus formed by displacement of quasi liquid from the contact zone is followed by a further growth of the meniscus volume. This will invalidate the condition *V*_1_ ≈ *V*_2_ and lead to a change of the meniscus shape. This dynamic change of the capillary bridge is discussed below.

### 3.3 Dynamic Nature of Microscopic Capillary Bridge

**Figure 5:**
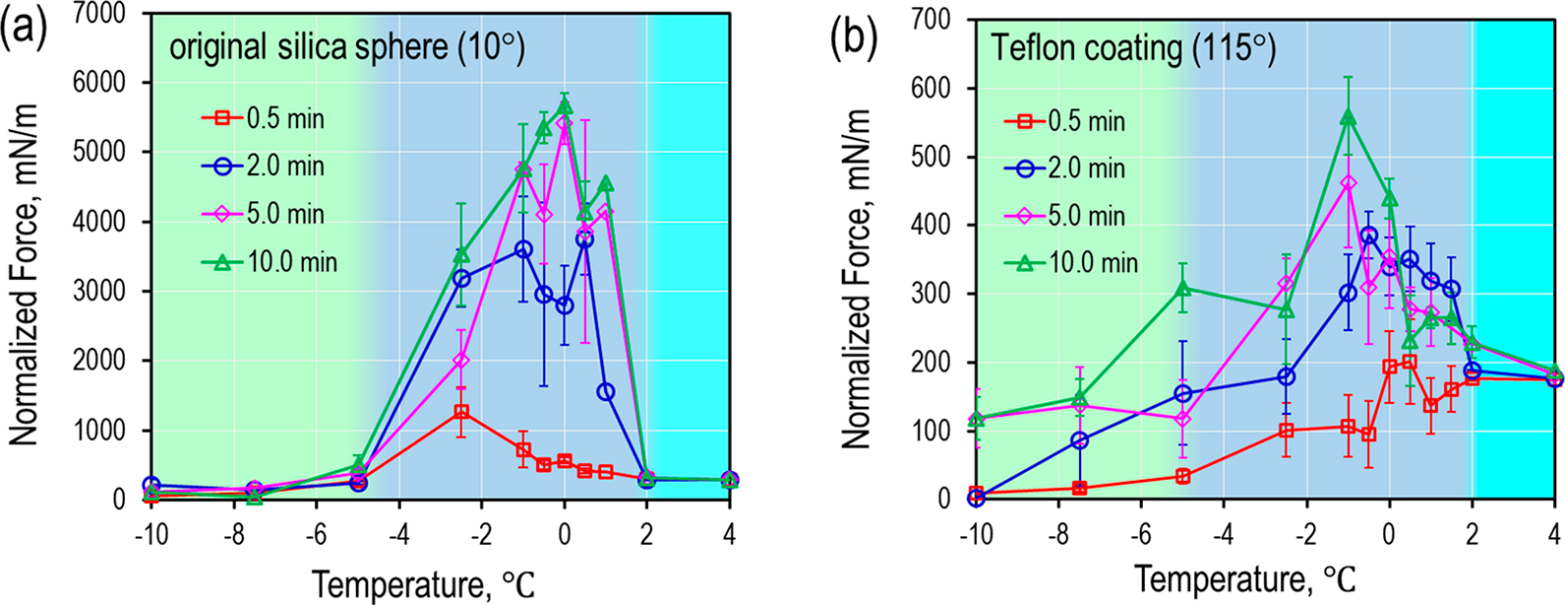
Time-dependent normalized adhesion force between a THF-*d*_8_ clathrate surface and an original silica sphere (a) or a Teflon-coated silica sphere (b). Both systems show a significant increase of adhesive force in the premelting region.

The normalized measured adhesive forces increase with increasing contact time (Figure 5). Although the measured forces exhibit different degrees of dependence on the contact time and the temperature, all curves show a similar “bell” shape with maximum values in the premelting region. This universal “bell” shape provides further evidence of the crucial role of the QLL in governing the adhesive forces of clathrate surfaces.

**Figure 6:**
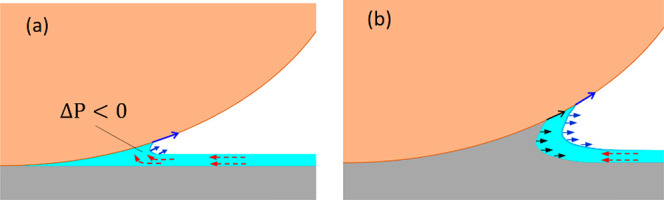
Proposed dynamic nature of the microscopic capillary bridge. Red dashed arrows indicate the flow of quasi liquid driven by negative Laplace pressure. Blue arrows indicate the expansion of the contact area by maintaining equilibrium thickness of QLL. Black arrows indicate the consolidation of the neck upon formation of additional clathrate. We assume that the QLL is in equilibrum with its bulk, and its thickness therefore remains constant.

Our model can explain the time-dependent increase of the measured adhesive forces in Figure 5. A large negative Laplace pressure in the capillary bridge (*F*_ΔP_ in Figure 4b) would drive a flow of quasi liquid into the neck and lead to the expansion of contact area and volume of the capillary bridge (Figure 6a). Assuming that the equilibrium thickness (δ) of the QLL has a fixed value for a given temperature, the increased volume of quasi-liquid at the neck would induce the formation of additional solid clathrate in the contact region (Figure 6b). This drives the formation of a molecular solid–solid (clathrate-sphere) contact that will lead to an additional adhesion. We ascribe the time-dependent increase of the measured adhesive force to the growing contribution of this solid–solid adhesion.

**Figure 7:**
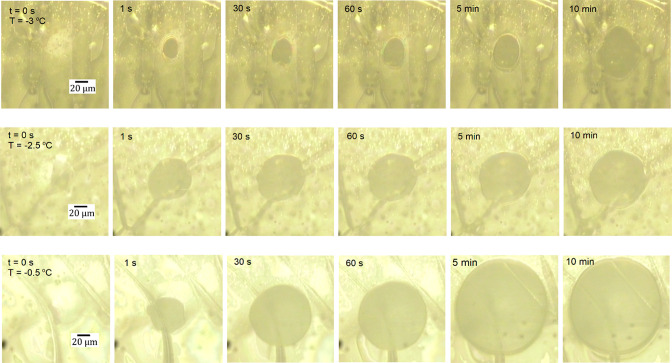
Expansion of the contact area between THF-*d*_8_ clathrate surface and original silica sphere as imaged on an inverted microscope. The thickness of the clathrate slab was 0.3 mm. Patterns in the images possibly arise from the boundaries between crystal domains.

We confirm the growth of the contact area with time using optical microscopy (Figure 7). The rate of contact area expansion increases with temperature. At −3 °C, the initial diameter (within time scale of seconds after the attachment) of the contact area is ≈20 μm which falls in the same order with the modeled diameter, i.e., 2 ×  = 11 μm in Figure 4c. At a given contact time, for example 30 s, the contact area increases with the temperature (Figure 7), possibly due to the growth of QLL thickness. At 1 °C, the expansion of the contact area and the formation of additional clathrate happens at a significant rate that can be viewed by the side-view optical camera (Figure S8).

## 4 Conclusions and Remarks

Conventional understanding attributes the adhesion between a clathrate surface and a solid surface to macroscopic water droplets sitting on either the clathrate surface or the solid surface. Such macroscopic water droplets, termed “free water”, form macroscopic capillary bridges and create capillary forces. This common mechanism of clathrate adhesion discussed in the literature is similar to the “dissociated region” in Figure 3a where a macroscopic capillary bridge is observed at, for example, 4 °C. However, the increase of the adhesive force in the premelting region and the role of the QLL-induced nano capillary bridge as the origin of such strong adhesion have never been reported before. We proved that the QLL-induced capillary attraction is the origin of the large adhesive force between clathrates and solid substrates.

The additional formation of clathrates at a capillary bridge has been discussed in the literature using the term of “sintering” effect.^45^ The “sintering” effect was considered to take place only upon addition of fresh water droplets. Again, those macroscopic water droplets could have created apparent capillary bridges between a clathrate and a counter surface.^45^ However, the consolidation effect explored in our study is induced by quasi-liquid flow and is governed by the interfacial premelting of the clathrate. No newly added “free water” is required nor involved in this consolidation effect discussed. Therefore, the present consolidation effect differs in nature from the “sintering” effect discussed in the literature.

The concept of surface coating for reducing clathrate adhesion have been considered by previous studies.^5,46−49^ We further proved this concept based on our theoretical and experimental evidence. The ultralow adhesion of the clathrate to rough hydrophobic (superhydrophobic-like) surfaces enables the removal of the attached clathrate particles by the viscous shear forces that are always present in hydrocarbon flow. For practical applications, however, the coating must have sufficient durability and robustness to withstand physical and chemical attritions (e.g., sand erosion) caused by actual conditions in pipelines.

## Supporting Information Available

Sphere coatings, contact angle measurement; force sensors; control of temperature and elimination of mechanical vibration; improved feature of our experimental design; equations for calculating the angle β; expansion and consolidation of contact area at 1 °C; solid sphere-clathrate surface and clathrate sphere-solid surface (PDF)
