# Supplementary material for: Clathrate Adhesion Induced by Quasi-Liquid Layer
Source: J Phys Chem C Nanomater Interfaces. 2021 Sep 16;125(38):21293–300. doi: 10.1021/acs.jpcc.1c06997 (PMC8488953; doi:10.1021/acs.jpcc.1c06997)
Supplement: Supplementary file 1 — jp1c06997_si_001.pdf [file jp1c06997_si_001.pdf]

Supporting Information

Clathrate Adhesion Induced by Quasi-Liquid Layer

Ngoc N. Nguyen[1,2,*], Rüdiger Berger[1,*], Michael Kappl[1], Hans-Jürgen Butt[1]

[1]Physics at Interfaces, Max Planck Institute for Polymer Research, Ackermannweg 10, 55128 Mainz, Germany

[2]School of Chemical Engineering, Hanoi University of Science and Technology, Dai Co Viet Street 1, Hanoi 100000, Vietnam

Corresponding authors E-mails: nguyenn@mpip-mainz.mpg.de (N.N.N.)

berger@mpip-mainz.mpg.de (R.B.)

This supplementary document provides additional information about the following aspects:

Section S1: Sphere Coatings, Contact Angle Measurement and Force Sensors.
Section S2: Control of Temperature and Elimination of Mechanical Vibration.
Section S3: Improved Feature of Our Experimental Design.
Section S4: Formulation of Equations for Calculating the Angle β.
Section S5: Expansion and Consolidation of Contact Area at 1 °C.
Section S6: Solid Sphere-Clathrate Surface and Clathrate Sphere-Solid Surface.

S1. Sphere Coatings, Contact Angle Measurement and Force Sensors

S1.1. Sphere Coatings

Original silica surface is very hydrophilic. We coated original silica spheres with different types of coating to achieve different contact angles. It is noted that we used two groups of spheres. The first one with radius of 0.8 mm is used as a probe for the force measurements. This sphere is referred to as "silica sphere". The second group includes micro silica spheres with mean diameter of 10 μm. This group is used for coating on the surface of the silica sphere to produce superhydrophobic surface. The second group is referred to as "microspheres".

Gold coating: A layer of gold was coated on the surface of silica spheres via physical vapour deposition (PVD) method and was implemented in a PVD facility (BAL-TEC, MED 020 Coating System). Metal gold was vaporized by argon plasma under a vacuum condition (absolute pressure of 10^{-5} at). The thickness of gold layer on silica sphere was 20 nm, which was estimated by the machine based on the plasma current and the working time. Gold coating is used in our study for fundamental understanding. We examined this layer after the force measurement experiments by means of Energy-dispersive X-ray (EDX) spectroscopy to see if the coated layer could come off the silica sphere. EDX results indicated the presence of gold throughout the surface and confirmed that the gold-coated layer stayed intact. Another point we like to discuss is that original silica spheres and gold-coated silica spheres might have certain degrees of surface roughness. However, the high wettability of these surfaces allows the QLL to fill the gaps between the

contacting asperities. Indeed, we only observed continuous contact areas between the spheres and the clathrate surfaces (Figure 7 in the manuscript).

Teflon coating: We coated the silica sphere with a film of Teflon using dip coating method. Teflon has been identified as having very low free surface energies and low adhesive properties.[1] Firstly, we prepared a solution that contained 4 wt.% Teflon AF1600 (Sigma Aldrich) in perfluoro-compound FC-43 (Sigma Aldrich). This solution is named here “Teflon solution”. The perfluoro-compound FC-43 served a solvent to dissolve Teflon AF1600. Then, a silica sphere was submerged in this solution for one minute. Upon taking the sphere out from the solution, a layer of the Teflon solution remained on the sphere surface. This liquid layer consolidated within minutes due to the evaporation of the solvent. The sphere with the layer of Teflon was then annealed at 160°C for 12 hours for complete removal of the solvent. It resulted in a stable film of Teflon covering the silica sphere. Our measurement based on a profiler technique (KLA-Tencor Stylus-Profiler model P7) showed that the Teflon film has a thickness of 400 nm.

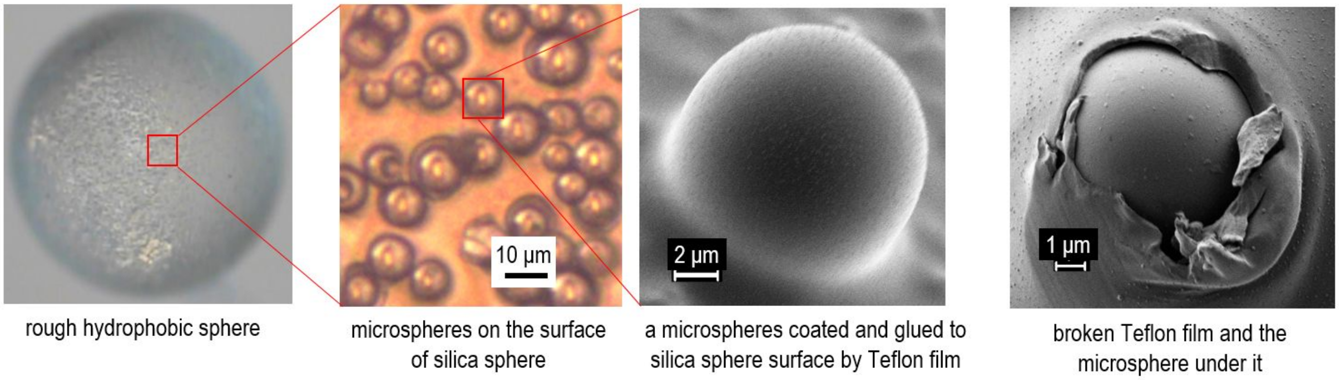

Figure S1. The surface of a rough hydrophobic sphere prepared and used in our experiments. Left to right: Optical image – microscope image – SEM (scanning electron microscopy) images.

Rough hydrophobic coating: We used the following method to coat a rough hydrophobic (superhydrophobic-like) layer on a silica sphere in replication of the natural structure of Lotus leaf. Firstly, microspheres with mean diameter of 10 μm was mixed with the aforementioned Teflon solution to produce a suspension of micro silica spheres in Teflon solution, concentration 5 wt.% (mass of microspheres per total mass of the suspension). The suspension was shaken vigorously before subjected to 30-minute sonication to facilitate the dispersion of the microspheres. Then, the coating was implemented through dip coating method: A silica sphere (radius of 0.8 mm) was submerged into this suspension for one minute. Upon taking the silica sphere out from the suspension, a layer of suspension with microspheres remained on the surface of the silica sphere, which was then consolidated upon the evaporation of the solvent and the microspheres were glued to the surface of the silica sphere. Finally, the sample was annealed at 160°C for 12 hours to remove the solvent completely and improve the mechanical strength of the Teflon film. Microspheres, which themselves were coated by Teflon film, were glued to the surface of silica sphere

(Figure S1). Hence, the silica sphere has a rough and hydrophobic surface, which exhibits a superhydrophobic-like feature. Our measurement based on a profiler technique (KLA-Tencor Stylus-Profiler model P7) showed that the Teflon film has a thickness of 400 nm. This thickness can also be observed visually by breaking the Teflon film and probing the Teflon layer using scanning electron microscopy (Figure S1).

S1.2. Contact Angle Measurement

Our model considers the contact angle between a solid sphere and the quasi liquid on the clathrate. It is not practically feasible to measure the contact angle created by quasi liquid. We therefore measured the contact angle of stoichiometric THF-d_8 solution on glass surfaces having identical coatings with the spheres. The measurement was implemented using a standard procedure on a commercial device (DataPhysics). The contact angle was derived from a built-in fitting software (Figure S2).

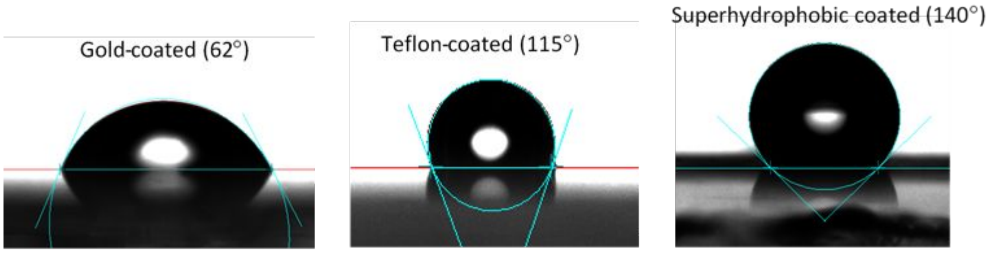

Figure S2. The contact angle of stoichiometric THF-d_8 solution on different coatings of glass surface.

S1.3. Force Sensors

We use hollow capillary tubes as mechanical force sensors adapted from our recent experiments.[2, 3] Two types of hollow capillary tubes are used, which had different geometries and sensitivities (Figure S3).

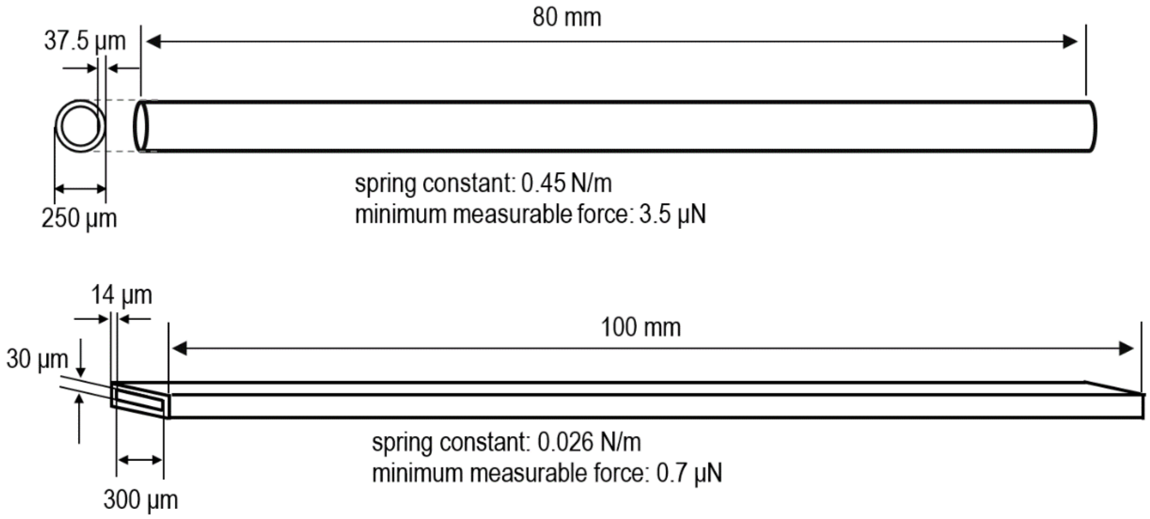

Figure S3. Force sensors based on hollow micro-capillary tubes used in this work.

S2. Control of Temperature and Elimination of Mechanical Vibration

A cold environment is required for experiments with clathrates. We built a "cold glove box" for our experiments by modifying a commercial freezer (GGM Gastro International) (Figure S4). The box offered an internal space of 2 m^3 for a cold environment. We built glove boxes through the front doors of the freezer (Figure S4). These glove boxes allow manipulating the experiments without the exchange between internal and external atmospheres. Otherwise, the migration of ambient air into the box led to serious condensation of moisture in relevant surfaces in the "cold glove box".

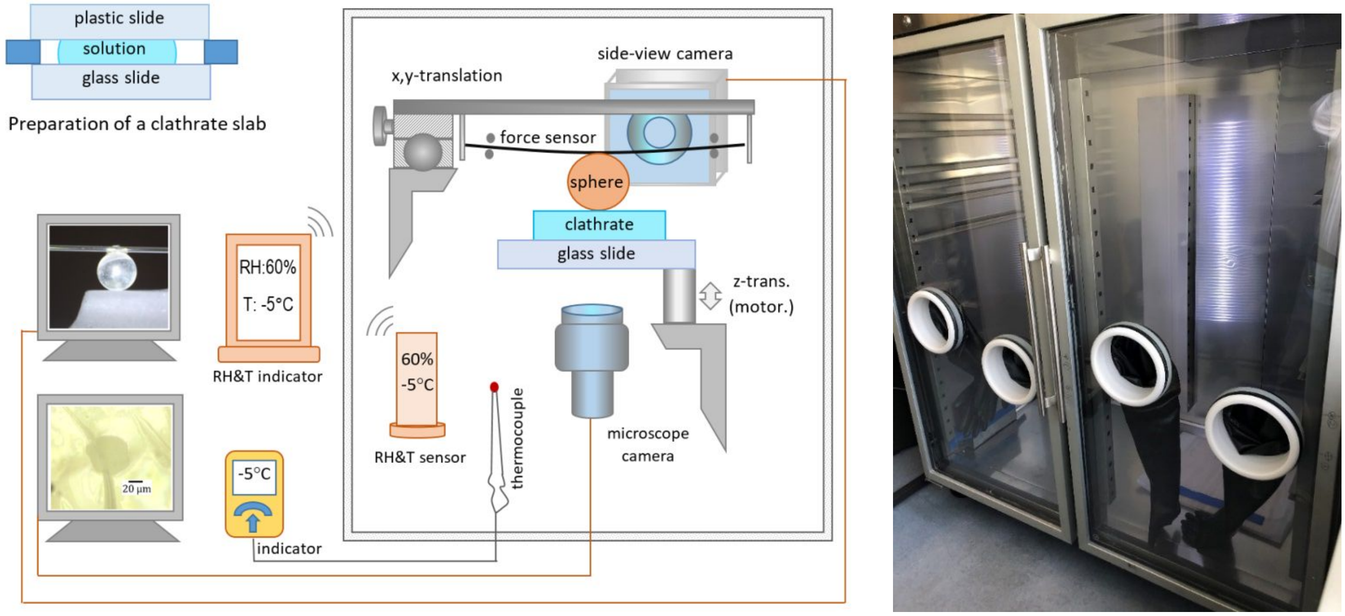

Figure S4. A homebuilt "cold glove box" for our experiments. This "cold glove box" was constructed based on modification of a commercial freezer. It is drawn as a squared box in the left and pictured in the right.

The premelting layer on clathrate surface is sensitive to the temperature. Moreover, the force measurement is sensitive to mechanical vibration. Therefore, maintenance of constant temperature and vibration-free conditions is a crucial requirement. No commercial freezer could satisfy these conditions. The temperature inside the freezer was found fluctuating periodically within ± 1°C around a set point following the on/off status of the cooling circle. In addition, the operation of the freezer produced significant vibration.

To avoid the vibration, we turned the freezer off during the force measurement. It however led to rapid increase of the temperature inside the "cold glove box", i.e. at a rate of 0.22 °C per minute. Hence, no constant-temperature conditions were possible. Our solution for this problem was to place a large number of water bottles in the free space in the "cold glove box". A total amount of 100 litres of water was used. This amount of water was frozen and cooled to -15°C. With this frozen water as a "cooling reservoir", the temperature inside the "cold glove box" increased much slower at a rate of 0.02°C per minute when the freezer was turned off. The longest run of our experiment is 10 minutes, i.e. the contact time was 10 minutes.

During 10 minutes, the temperature was changed by 0.2°C, which fell within the accuracy of the temperature measurement (± 0.1°C). Therefore, the "cooling reservoir" provided an excellent measure to achieve constant-temperature and vibration-free conditions for the measurement of the adhesive force.

In addition, it offered an effective way to perform the temperature-dependent experiments. Firstly, we ran the freezer for a day to stabilize the temperature at -15°C, i.e. freezing the water in the bottles and cooling the frozen water to -15°C. Secondly, we turned the freezer off. While the temperature inside the "cold glove box" increased slowly, we carried out the measurement at each temperature that the system passed through. For example, we measured the force at -10°C, waited for the temperature to reach -7.5°C to carry out another measurement at this temperature, then waited for -5.0°C and carried out another measurement, and so forth. Slow increase of the temperature in the "cold glove box" ensured that every measurement was conducted at a quasi-constant temperature.

S3. Improved Feature of Our Experimental Design

Our experimental design enables accurate measurements of the capillary force and quantify the dynamic effects of the microscopic capillary bridge on the capillary force. By allowing the two ends of cantilever to move flexibly in a horizontal direction, we are able to maintain a static contact between the sphere and clathrate surface until the detachment occurs. This is a minor but elegant modification compared to a classical setup widely used in previous studies (Figure S5). The latter is based on a partially fixed cantilever whose one end is firmly fixed to a holder and the other end is bound to a particle. The classical setup leads to an inherent drawback: the sphere continuously slides (Δx) and rotates ($\Delta\varphi$) relatively to the clathrate surface (Figure S5). The resulting dragging force destroys any consolidation processes. In fact, forces measured by our new design are orders of magnitude larger than the ones measured by the classical design under identical experimental conditions (Figure S5).

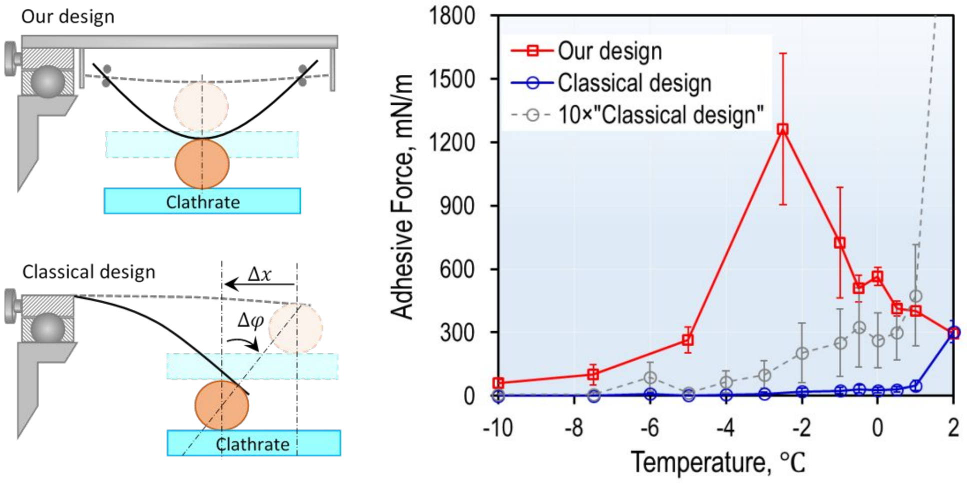

Figure S5. Comparative measurements of the capillary force between a clathrate and a silica sphere using our setup and the a classical design under identical experimental conditions.

S4. Formulation of Equations for Calculation of the Angle β

Here, we establish equations for calculating the angle β in Figure S6. This angle is needed for the calculation of the adhesive force in the main manuscript. When the sphere approaches the clathrate surface, it displaces a volume of quasi-liquid (V_1, yellow region) and leads to formation of a capillary bridge (V_2, green region). These volumes are equal.

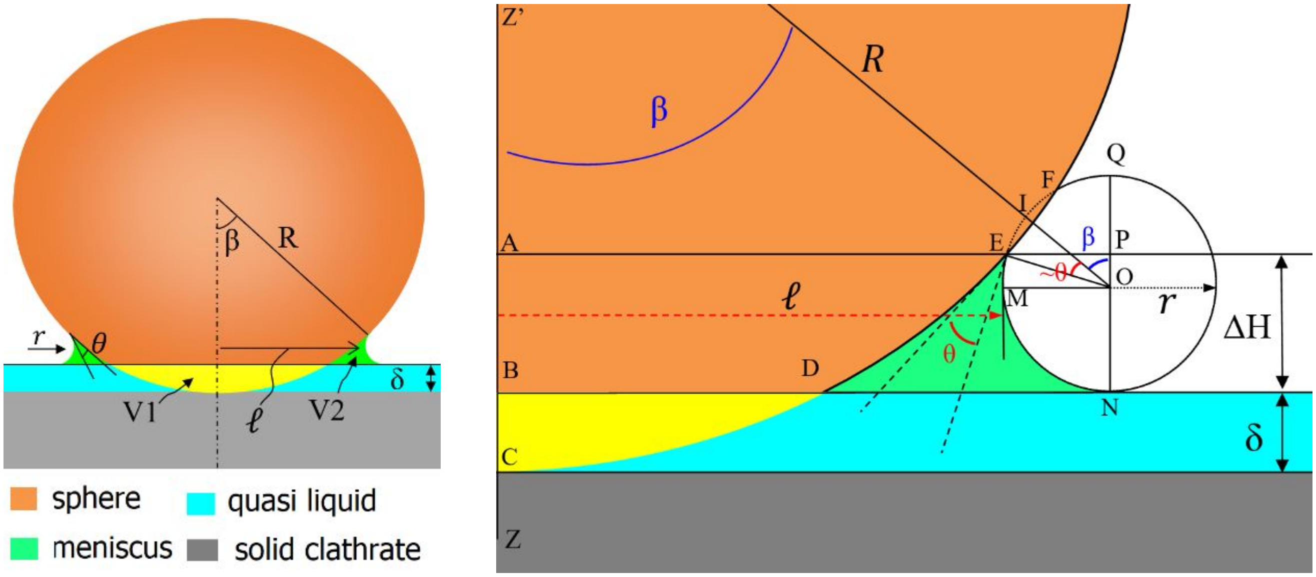

Figure S6. Conceptualization of a capillary bridge formation between a clathrate surface and a solid sphere.

$$V_1 = V_2 \qquad \text{(S.1)}$$

V_1 is the volume of a spherical cap (yellow region). This volume is:

$$V_1 = \pi\delta^2 \times \left(R - \frac{\delta}{3}\right) \qquad \text{(S.2)}$$

V_2 is related to the volumes of other segments (Figure S7) as described by Eq. (S.3).

$$V_3 = V_2 + V_4 + V_5 \qquad \text{(S.3)}$$

V_3 is the volume of a cylinder formed by rotating rectangle ABNP about zz' pivot (Figure S7). We have:

$$V_3 = \pi(\ell + r)^2 \times \Delta H \qquad \text{(S.4)}$$

$$\Delta H = \overline{\text{ON}} + \overline{\text{OP}} = r + r\cos(\beta + \theta) = r[1 + \cos(\beta + \theta)] \qquad \text{(S.5)}$$

We also have:

$$\Delta H = \text{R}(1 - \cos\beta) - \overline{\text{IL}} - \overline{\text{BC}} = R(1 - \cos\beta) - r \times \sin\theta\sin\beta - \delta \qquad \text{(S.6)}$$

Combining Eq. (S.5) and Eq. (S.6) yields to:

$$r = \frac{R(1 - \cos\beta) - \delta}{1 + \cos(\beta + \theta) + \sin\theta\sin\beta} \qquad \text{(S.7)}$$

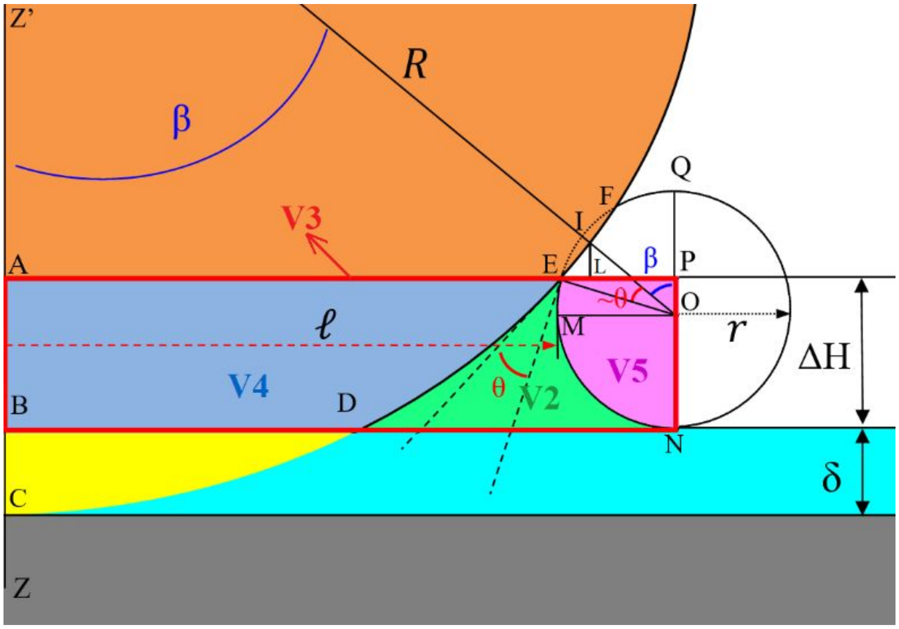

Figure S7. Analysis of the geometry of the capillary bridge.

Thus, Eq. (S.6) becomes:

$$\Delta H = R(1-\cos\beta) - \delta - \frac{R(1-\cos\beta)-\delta}{1+\cos(\beta+\theta)+\sin\theta\sin\beta}\sin\theta\sin\beta \tag{S.8}$$

We also have:

$$\ell = R\sin\beta - r(1-\sin\beta) \tag{S.9}$$

Substituting Eq. (S.7) into Eq. (S.9) gives:

$$\ell = R\sin\beta - \frac{R(1-\cos\beta)-\delta}{1+\cos(\beta+\theta)+\sin\theta\sin\beta}(1-\sin\beta) \tag{S.10}$$

Substituting Eqs. (S.7),(S.8) and (S.10) into Eq. (S.4), we have:

$$V_3 = \pi\left[R\sin\beta - \frac{R(1-\cos\beta)-\delta}{1+\cos(\beta+\theta)+\sin\theta\sin\beta}(1-\sin\beta) + \frac{R(1-\cos\beta)-\delta}{1+\cos(\beta+\theta)+\sin\theta\sin\beta}\right]^2 \times \left[R(1-\cos\beta) - \delta - \frac{R(1-\cos\beta)-\delta}{1+\cos(\beta+\theta)+\sin\theta\sin\beta}\sin\theta\sin\beta\right]$$

(S.11)

Simplifying (S.11) gives:

$$V_3 = \pi\left[\left(R - \frac{R(1-\cos\beta)-\delta}{1+\cos(\beta+\theta)+\sin\theta\sin\beta}\right)\sin\beta\right]^2\left[R(1-\cos\beta) - \delta - \frac{R(1-\cos\beta)-\delta}{1+\cos(\beta+\theta)+\sin\theta\sin\beta}\sin\theta\sin\beta\right] \tag{S.12}$$

V_4 is the volume of a truncated spherical cap shown in blue (Figure S7).

$$V_4 = \pi\overline{AC}^2 \times \left(R - \frac{\overline{AC}}{3}\right) - \pi\overline{BC}^2 \times \left(R - \frac{\overline{BC}}{3}\right) \tag{S.13}$$

$$V_4 = \pi(\Delta H + \delta)^2 \times \left(R - \frac{\Delta H + \delta}{3}\right) - \pi\delta^2 \times \left(R - \frac{\delta}{3}\right) \tag{S.14}$$

Substituting (S.8) into (S.14) we have:

$$V_4 = \pi\Big[R(1-\cos\beta) - \delta - \frac{R(1-\cos\beta)-\delta}{1+\cos(\beta+\theta)+\sin\theta\sin\beta}\sin\theta\sin\beta + \delta)\Big]^2 \times [R - \frac{R(1-\cos\beta) - \delta - \frac{R(1-\cos\beta)-\delta}{1+\cos(\beta+\theta)+\sin\theta\sin\beta}\sin\theta\sin\beta + \delta}{3}] - \pi\delta^2 \times (R - \frac{\delta}{3}) \quad \text{(S.15)}$$

Simplifying Eq. (S.15) gives:

$$V_4 = \pi\Big[R(1-\cos\beta) - \frac{R(1-\cos\beta)-\delta}{1+\cos(\beta+\theta)+\sin\theta\sin\beta}\sin\theta\sin\beta)\Big]^2 \times \left[R - \frac{R(1-\cos\beta) - \frac{R(1-\cos\beta)-\delta}{1+\cos(\beta+\theta)+\sin\theta\sin\beta}\sin\theta\sin\beta}{3}\right] - \pi\delta^2 \times (R - \frac{\delta}{3}) \quad \text{(S.16)}$$

V_5 is the volume formed by rotating the pink area about zz' pivot (Figure S7). It is approximately equal to:

$$V_5 = S \times 2\pi(\ell + r) \quad \text{(S.17)}$$

Where S is the area of the pink region in Figure S7. This area is

$$S = \frac{1}{2}\Big\{\pi r^2 - \frac{r^2}{2}[2(\beta+\theta) - \sin(2(\beta+\theta))]\Big\} \quad \text{(S.18)}$$

$$S = \frac{1}{4}r^2 \quad \text{(S.19)}$$

Substituting Eq. (S.7) into Eq. (S.13) gives:

$$S = \frac{1}{4}\Big[\frac{R(1-\cos\beta)-\delta}{1+\cos(\beta+\theta)+\sin\theta\sin\beta}\Big]^2 \times [2\pi - 2(\beta+\theta) + \sin(2(\beta+\theta))] \quad \text{(S.20)}$$

Substituting Eq. (S.7), Eq. (S.10) and Eq. (S.20) into Eq. (S.17) gives:

$$V_5 = \frac{1}{4}\Big[\frac{R(1-\cos\beta)-\delta}{1+\cos(\beta+\theta)+\sin\theta\sin\beta}\Big]^2 \times [2\pi - 2(\beta+\theta) + \sin(2(\beta+\theta))] \times 2\pi \Big[R\sin\beta - \frac{R(1-\cos\beta)-\delta}{1+\cos(\beta+\theta)+\sin\theta\sin\beta}(1-\sin\beta) + \frac{R(1-\cos\beta)-\delta}{1+\cos(\beta+\theta)+\sin\theta\sin\beta}\Big] \quad \text{(S.21)}$$

Simplifying Eq. (S.15) gives:

$$V_5 = \frac{1}{2}\pi\sin\beta\Big[\frac{R(1-\cos\beta)-\delta}{1+\cos(\beta+\theta)+\sin\theta\sin\beta}\Big]^2[2\pi - 2(\beta+\theta) + \sin(2(\beta+\theta))]\Big[R + \frac{R(1-\cos\beta)-\delta}{1+\cos(\beta+\theta)+\sin\theta\sin\beta}\Big] \quad \text{(S.22)}$$

Substituting Eq. (S.2), Eq. (S.12), Eq. (S.16) and Eq. (S.22) into Eq. (S.3), we have:

$$\pi\Big[\Big(R - \frac{R(1-\cos\beta)-\delta}{1+\cos(\beta+\theta)+\sin\theta\sin\beta}\Big)\sin\beta\Big]^2\Big[R(1-\cos\beta) - \delta - \frac{R(1-\cos\beta)-\delta}{1+\cos(\beta+\theta)+\sin\theta\sin\beta}\sin\theta\sin\beta\Big] = \pi\delta^2\Big(R - \frac{\delta}{3}\Big) + \pi \Big[R(1-\cos\beta) - \frac{R(1-\cos\beta)-\delta}{1+\cos(\beta+\theta)+\sin\theta\sin\beta}\sin\theta\sin\beta)\Big]^2\Big[R - \frac{R(1-\cos\beta) - \frac{R(1-\cos\beta)-\delta}{1+\cos(\beta+\theta)+\sin\theta\sin\beta}\sin\theta\sin\beta}{3}\Big] - \pi\delta^2(R - \frac{\delta}{3}) + \frac{1}{2} \pi\sin\beta\Big[\frac{R(1-\cos\beta)-\delta}{1+\cos(\beta+\theta)+\sin\theta\sin\beta}\Big]^2[2\pi - 2(\beta+\theta) + \sin(2(\beta+\theta))]\Big[R + \frac{R(1-\cos\beta)-\delta}{1+\cos(\beta+\theta)+\sin\theta\sin\beta}\Big] \quad \text{(S.23)}$$

Simplifying Eq. (S.23) yields to:

$$\Big[\Big(1 - \frac{1-\cos\beta - \delta/R}{1+\cos(\beta+\theta)+\sin\theta\sin\beta}\Big)\sin\beta\Big]^2\Big[1 - \cos\beta - \frac{\delta}{R} - \frac{1-\cos\beta - \delta/R}{1+\cos(\beta+\theta)+\sin\theta\sin\beta}\sin\theta\sin\beta\Big]$$

$$= \left[(1-\cos\beta) - \frac{1-\cos\beta-\delta/R}{1+\cos(\beta+\theta)+\sin\theta\sin\beta}\sin\theta\sin\beta)\right]^2\left[1-\frac{(1-\cos\beta)-\frac{(1-\cos\beta)-\delta/R}{1+\cos(\beta+\theta)+\sin\theta\sin\beta}\sin\theta\sin\beta}{3}\right]$$

$$+\frac{1}{2}\sin\beta\left[\frac{1-\cos\beta-\delta/R}{1+\cos(\beta+\theta)+\sin\theta\sin\beta}\right]^2[2\pi-2(\beta+\theta)+\sin(2(\beta+\theta))]\left[1+\frac{1-\cos\beta-\delta/R}{1+\cos(\beta+\theta)+\sin\theta\sin\beta}\right] \quad \text{(S.24)}$$

Eq. (S.24) contains only β as a variable. This equation is the Eq. (4) in the main manuscript.

S5. Expansion and Consolidation of Contact Area at 1 °C

At 1°C, we observed an extraordinary phenomenon. Shortly after a silica sphere contacted the clathrate surface, an apparent capillary bridge forms at the contact area (marked by the red oval, Figure S8). The clathrate reforms in the neck and then grows upward on the surface of the silica sphere. We explain this phenomenon using the sketch in Figure S8. Generally, water has a tendency of spreading over a silica surface, i.e. the blue arrow in the sketch, due to the hydrophilic nature of silica. For bulk liquid water, the upward motion of the three-phase contact line is hindered by the gravity of the water that tends to pull the three-phase contact line downward. For QLL, this effect of gravity might be negligible due to its insignificant volume. Therefore, the wetting of the QLL is governed exclusively by the wettability of the solid surface. When the QLL spreads, the inner layer reforms clathrate and consolidates on the sphere surface. The external layer remains liquid and serves as a transport channel to supply the solution for the "climbing" growth of the clathrate on the silica sphere (Figure S8).

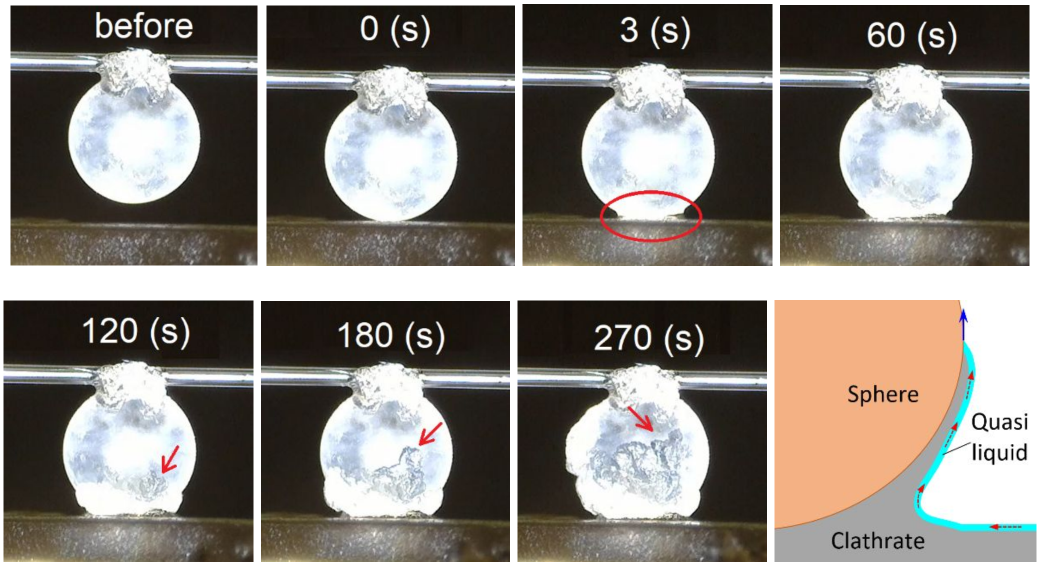

Figure S8. Expansion and consolidation of the contact area at 1 °C. The spreading of quasi-liquid and subsequent reformation of clathrate induce a "climbing" growth of clathrate on the sphere.

S6. Solid Sphere-Clathrate Surface and Clathrate Sphere-Solid Surface

We investigate a solid sphere interacting with a clathrate surface. The system relevant to industrial applications involves a clathrate particle interacting with a solid surface. We used the earlier system to avoid the difficult preparation of smooth spherical clathrate particles associated in the later system. Here, we demonstrate that these two systems exhibit identical adhesive forces.

<table>
<tr>
<td>Model</td>
<td>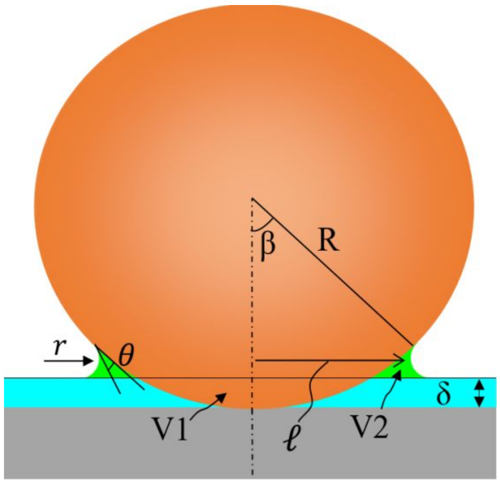

System: Solid sphere - clathrate surface
(the present study)</td>
<td>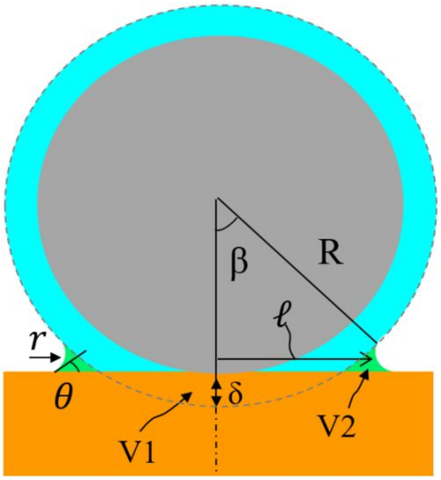

Clathrate sphere - solid surface
(our recent study, ref.[4])</td>
</tr>
<tr>
<td>Capillary force equations</td>
<td>$F_{ca} = -2\pi\ell\gamma - \pi\ell^2\gamma\left(\frac{1}{r} - \frac{1}{l}\right)$
$r = \frac{R(1-\cos\beta)-\delta}{1+\cos(\beta+\theta)+\sin\theta\times\sin\beta}$
$\ell = R\sin\beta - r(1-\sin\beta)$</td>
<td>$F_{ca} = -2\pi\ell\gamma - \pi\ell^2\gamma\left(\frac{1}{r} - \frac{1}{l}\right)$
$r = \frac{R(1-\cos\beta)-\delta}{\cos\beta+\cos\theta}$
$\ell = R\sin\beta - \frac{[R(1-\cos\beta)-\delta]\times(1-\sin\beta)}{\cos\beta+\cos\theta}$</td>
</tr>
<tr>
<td>Normalized force</td>
<td>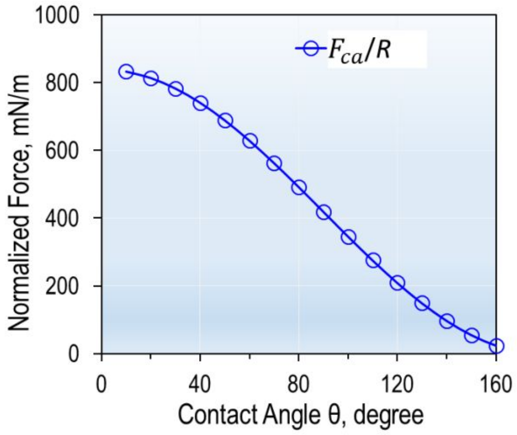
</td>
<td>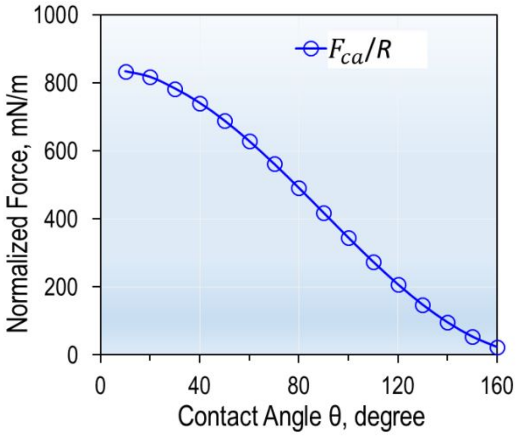
</td>
</tr>
</table>

Reference

(1) Drummond, C. J.; Georgaklis, G.; Chan, D. Y. C.,Fluorocarbons: Surface Free Energies and van der Waals Interaction. *Langmuir* **1996**, *12*, 2617-2621.

(2) Gao, N.; Geyer, F.; Pilat, D. W.; Wooh, S.; Vollmer, D.; Butt, H.-J.; Berger, R. How Drops Start Sliding over Solid Surfaces. *Nat. Phys.* **2018**, *14*, 191-196.

(3) Geyer, F.; D'Acunzi, M.; Sharifi-Aghili, A.; Saal, A.; Gao, N.; Kaltbeitzel, A.; Sloot, T.-F.; Berger, R.; Butt, H.-J.; Vollmer, D. When and How Self-cleaning of Superhydrophobic Surfaces Works. *Sci. Adv.* **2020**, *6*, 9727.

(4) Nguyen, N. N.; Berger, R.; Butt, H.-J. Premelting-Induced Agglomeration of Hydrates: Theoretical Analysis and Modeling. *ACS Appl. Mater. Interfaces* **2020**, *12*, 14599-14606.
